# Rare Variants Associated with Arrhythmogenic Cardiomyopathy: Reclassification Five Years Later

**DOI:** 10.3390/jpm11030162

**Published:** 2021-02-26

**Authors:** Marta Vallverdú-Prats, Mireia Alcalde, Georgia Sarquella-Brugada, Sergi Cesar, Elena Arbelo, Anna Fernandez-Falgueras, Mónica Coll, Alexandra Pérez-Serra, Marta Puigmulé, Anna Iglesias, Victoria Fiol, Carles Ferrer-Costa, Bernat del Olmo, Ferran Picó, Laura Lopez, Paloma Jordà, Ana García-Álvarez, Coloma Tirón de Llano, Rocío Toro, Simone Grassi, Antonio Oliva, Josep Brugada, Ramon Brugada, Oscar Campuzano

**Affiliations:** 1 Cardiovascular Genetics Center, University of Girona—IdIBGi, 17190 Girona, Spain; mvallverdu@gencardio.com (M.V.-P.); malcalde@gencardio.com (M.A.); afernandez@gencardio.com (A.F.-F.); mcoll@gencardio.com (M.C.); aperez@idibgi.org (A.P.-S.); mpuigmule@gencardio.com (M.P.); annai@brugada.org (A.I.); cferrer@gencardio.com (C.F.-C.); bdelolmo@gencardio.com (B.d.O.); ferran.pico@gencardio.com (F.P.); llopez@gencardio.com (L.L.); 2 Centro Investigación Biomédica en Red de Enfermedades Cardiovasculares (CIBERCV), 28029 Madrid, Spain; EARBELO@clinic.cat (E.A.); paloma.jorda.b@gmail.com (P.J.); ANAGARCI@clinic.cat (A.G.-Á.); jbrugada@clinic.cat (J.B.); 3 Medical Science Department, School of Medicine, University of Girona, 17071 Girona, Spain; georgia@brugada.org; 4 Arrhythmias Unit, Hospital Sant Joan de Déu, University of Barcelona, 08950 Barcelona, Spain; sergi.cesar@gmail.com (S.C.); jvfiolramis@gmail.com (V.F.); 5 Arrhythmias Unit, Hospital Clinic de Barcelona, University of Barcelona, 08036 Barcelona, Spain; 6 Cardiology Service, Hospital Dr. Josep Trueta, University of Girona, 17007 Girona, Spain; colomatiron@gmail.com; 7 Medicine Department, School of Medicine, 11003 Cadiz, Spain; rociotorogreen@gmail.com; 8 Institute of Public Health, Section Legal Medicine, Catholic University, 00153 Rome, Italy; simone.grassi@unicatt.it (S.G.); antonio.oliva@unicatt.it (A.O.)

**Keywords:** sudden cardiac death, arrhythmogenic cardiomyopathy, genetics, rare variants, reclassification

## Abstract

Genetic interpretation of rare variants associated with arrhythmogenic cardiomyopathy (ACM) is essential due to their diagnostic implications. New data may relabel previous variant classifications, but how often reanalysis is necessary remains undefined. Five years ago, 39 rare ACM-related variants were identified in patients with features of cardiomyopathy. These variants were classified following the American College of Medical Genetics and Genomics’ guidelines. In the present study, we reevaluated these rare variants including novel available data. All cases carried one rare variant classified as being of ambiguous significance (82.05%) or likely pathogenic (17.95%) in 2016. In our comprehensive reanalysis, the classification of 30.77% of these variants changed, mainly due to updated global frequencies. As in 2016, nowadays most variants were classified as having an uncertain role (64.1%), but the proportion of variants with an uncertain role was significantly decreased (17.95%). The percentage of rare variants classified as potentially deleterious increased from 17.95% to 23.07%. Moreover, 83.33% of reclassified variants gained certainty. We propose that periodic genetic reanalysis of all rare variants associated with arrhythmogenic cardiomyopathy should be undertaken at least once every five years. Defining the roles of rare variants may help clinicians obtain a definite diagnosis.

## 1. Introduction

Arrhythmogenic cardiomyopathy (ACM) is a rare disorder characterized by progressive replacement of the myocardium by fibrofatty tissue, and this myocyte disorganization increases the risk for ventricular arrhythmias and sudden cardiac death [1,2]. Tissue substitution occurs predominantly in the right ventricle, but biventricular forms have also been reported [3]. Moreover, isolated forms affecting only the left ventricle have also been published [4]. Diagnosis of ACM does not rely on a single gold standard test but is achieved using a scoring system proposed in 1994 by Task Force Criteria (TFC), encompassing familial and genetic factors, electrocardiogram (ECG) abnormalities, and structural/functional ventricular alterations [5,6]. In 2010, a revision of the TFC was proposed to incorporate new knowledge and technology to improve diagnostic sensitivity while maintaining diagnostic specificity [7]. This is important because correct diagnosis prevents over- or under-treatment and reduces the morbidity and mortality of ACM patients [8]. Also, there is evidence that differences in the propensity for life-threatening arrhythmias, left ventricular dysfunction, and heart failure can be explained by gene-specific alterations [9].

A definite genetic variant classified as pathogenic represents a major criterion for ACM diagnosis in the TFC [10]. Currently, comprehensive genetic analysis allows identification of a genetic cause in up to 60% of ACM cases [11]. Although there are several genes reported to be associated with ACM, most ACM patients carry pathogenic alterations in genes encoding desmosomal proteins, with *PKP2* being the main gene currently associated with ACM [12]. However, genetic diagnosis is challenging in ACM families because the clinical role of rare variants located in known genes is not always clear due to a lack of conclusive data as well as overlap of causal genes with other inherited cardiomyopathies [10,12,13]. This ambiguity impedes inclusion of genetic results in a definite diagnosis of ACM. The American College of Medical Genetics and Genomics (ACMG) standards and guidelines have structured standard terminology for classifying sequence variants using available evidence weighted according to a system developed through expert opinion, work-group consensus, and community input [14]. Accurate genetic interpretation following ACMG recommendations facilitates clinical translation of rare genetic variants and more individualized clinical management of patients [15].

Continuous improvement in the genetic data concerning rare variants may modify previous classifications and thus alter patient diagnosis and treatment, prenatal diagnosis or pre-implantation genetic diagnosis [16], and screening of at-risk family members [13,16]. To date, few studies have focused on proper genetic reinterpretation of available data before clinical translation [17,18]. These limited studies have focused on inherited arrhythmogenic diseases but have not addressed how often reanalysis is necessary in ACM-related genes. Our study aims to clarify this crucial point, facilitating accurate genetic classification, clinical diagnosis, and the adoption of personalized measures in families afflicted by ACM.

## 2. Materials and Methods

### 2.1. Cohort

We reanalysed and reinterpreted 39 rare variants identified in 2016 from 39 patients that presented features of ACM (definite, borderline or possible diagnosis following TFC). These rare variants were originally classified as pathogenic (P), likely pathogenic (LP), or variants of unknown significance (VUS) following ACMG recommendations [14]. Variants classified as likely benign (LB) and/or benign (B) in 2016 were not reanalysed because their global frequencies in 2016 were >1%, and they were already reported to be definitively non-causative in ACM. Genetic analysis was approved by the ethics committee of Hospital Josep Trueta (Girona, Spain) following the World Medical Association Declaration of Helsinki. Clinical and genetic data concerning all patients were kept anonymous. Written informed consent was obtained from all patients included in the study before genetic analysis.

### 2.2. Genetic Analysis

Genomic DNA was extracted from whole blood, and its concentration and purity were determined. Comprehensive genetic analysis was performed, including all genes associated with ACM in 2016 and all isoforms described in Ensembl 75 (www.ensembl.org/) linked to RefSeq code (www.ncbi.nlm.nih.gov/refseq/) or CCDS (www.ncbi.nlm.nih.gov/CCDS/). Sequence data coordinates were based on the UCSC human genome version hg19 (NCBI GRCh37 build). Secondary, bioinformatic analysis included adaptor and low-quality base-trimming of the FASTQ files. Variant calling from the cleaned BAM files was performed with SAMtools v.1.2 and an ad hoc developed script. The final annotation steps provided information included in public databases. Non-common (minor allele frequency [MAF] <1%) genetic variants were confirmed by Sanger sequencing. Exons and exon–intron boundaries of each gene were amplified in both directions with posterior analysis, comparing obtained results with the reference sequence from hg19.

Identified rare variants were compared with with HapMap (www.hapmap.ncbi.nlm.nih.gov/), the 1000 Genomes Project (www.1000genomes.org/), the Exome Variant Server (EVS) (www.evs.gs.washington.edu/EVS/) in 2016, and the Genome Aggregation Database (gnomAD) (www.gnomad.broadinstitute.org/) in 2021. All rare variants were described following HGVS (www.hgvs.org/) and consulted in ClinGen (www.clinicalgenome.org/), VarSome (www.varsome.com/), the SCD-associated Variants Annotation Database (SVAD) (www.svad.mbc.nctu.edu.tw/), CardioClassifier (www.cardioclassifier.org/), InterVar (www.wintervar.wglab.org/), CardioVAI (www.cardiovai.engenome.com/ and CardioBoost (www.cardiodb.org/cardioboost/).

### 2.3. Data

An exhaustive review of the literature concerning each variant was performed through January 2021. Data was collected from HGMD (www.hgmd.org), ClinVar (www.ncbi.nlm.nih.gov/clinvar/intro/), the National Center for Biotechnology Information SNP database (www.ncbi.nlm.nih.gov/SNP), Index Copernicus (www.en.indexcopernicus.com), Google Scholar (www.scholar.google.es), Springer Link (www.link.springer.com), Science Direct (www.sciencedirect.com), Excerpta Medica Database (www.elsevier.com/solutions/embase-biomedical-research), and IEEE Xplore Digital Library (www.ieeexplore.ieee.org/Xplore/home.jsp).

### 2.4. Classification

In 2016, rare variants were classified following the same ACMG standards currently in use [14]. In 2021, variants were classified following the updated ACMG standards [19,20]. The PM2 item in the ACMG classification was calculated based on [21] to find the threshold of the rare variant frequency.

The classification “high degree of pathogenicity” should only be used for rare variants in genes in which loss of function is a well-established disease mechanism [22]. Genetic data were independently evaluated and classified by six experts (three cardiologists and three clinical geneticists). All investigators discussed and agreed on final classification of all variants to avoid bias.

## 3. Results

**Figure 1:**
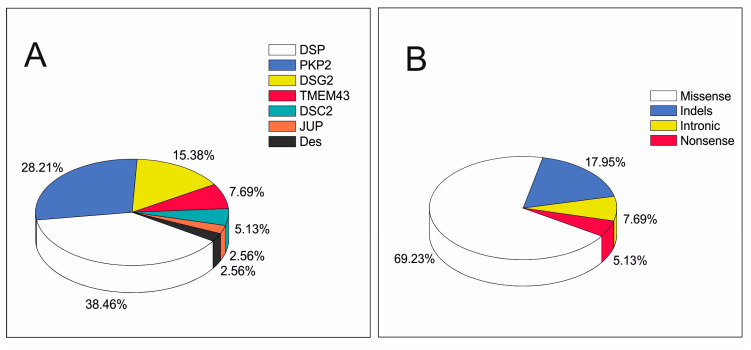
Rare variant distribution in genes and mutation types. (**A**) Localization of the 39 evaluated variants in arrhythmogenic cardiomyopathy (ACM)-causal genes. (**B**) Distribution of mutation types of the 39 evaluated variants.

We reinterpreted 39 rare variants in ACM related genes identified in 39 non-related Caucasian patients with features of ACM in 2016 (9 definite and 30 possible diagnosis in 2016. These rare variants were mainly located in desmosomal genes (35/39 = 89.75%): 11 in *PKP2*, 15 in *DSP*, six in *DSG2*, two in *DSC2* and one in *JUP*. There were also four rare variants in non-desmosomal genes (4/39 = 10.25%): three in *TMEM43* and one in *DES* (Figure 1A). Most rare variants were missense (27, 69.23%), and there were also seven indels (17.94%), three intronic variants (7.69%), and two nonsense variants (5.13%) (Figure 1B).

After a comprehensive analysis, 12 of the 39 rare variants (30.77%) were reclassified (Table 1). Most of the rare variants were classified as variants of unknown significance (VUS) in 2016 (32 rare variants, 82.05%) and in the current reclassification the number has been significantly reduced (25 rare variants, 64.10%). Thus, the number of variants classified as VUS was reduced by 17.95% in our reassessment.

**Figure 2:**
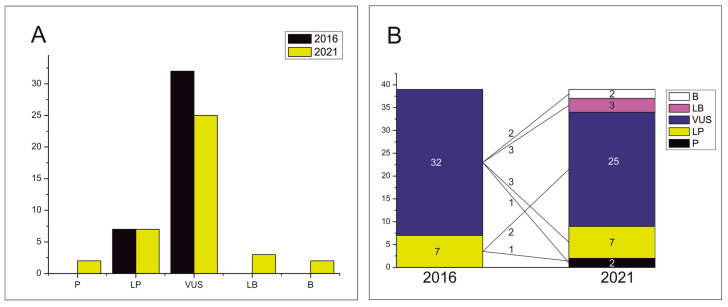
Distribution of the 39 revaluated variants into five groups of variant significance *. (**A**) Comparison of the number of variants in each group of significance. (**B**) Comparison of the number of variants between 2016 and 2021 classifications. * Five groups of variant significance: P = pathogenic; LP = likely pathogenic; VUS =variant of uncertain significance; LB = likely benign, and B = benign.

Of the nine reclassified VUS variants, two were now classified as benign, three as likely benign (LB) and four as likely pathogenic (LP). In 2016 we identified seven likely pathogenic (LP) variants (17.94%), but only four remained LP five years after. Two of the 2016 LP variants changed to VUS and one to P (Figure 2).

Importantly, 10 out of 12 (83.33%) reclassified variants gained certainty upon reclassification. Among likely pathogenic mutations, 37.5% (3/8) qualified as predicted null variants in a gene where loss of function is a known mechanism of disease (very strong criteria-PVS1). Only one missense variant (variant 4 DSG2;c.146G>A) was classified as P due to the sum of different criteria and with several strong supporting clinical reports available (very strong criteria-PP5) (Table 2). On the other hand, all LB and B variants (in desmosomal genes) were frequent enough to qualify as strong criteria in the population data (BA1/BS1) (Table 2). In 2016, the ExAC database showed a total of 31 rare variants without frequency data in the global population (79.48%). Updated global frequencies in the gnomAD database showed that the absolute number of rare variants found in the general population increased in comparison to 2016, and only 18 rare variants still remain unidentified in the general population (46.15%).

## 4. Discussion

In our study, 39 rare variants in ACM-related genes were identified in 39 non-related Caucasian patients with features of ACM five years ago. Using currently available data, we updated the 2016 classifications of 30.77% of the rare variants. Importantly, 83.33% of these relabelled rare variants gained certainty in their significance. Our results support periodic evaluation of rare variants associated with ACM at least once every five years as this may directly impact final diagnosis as described in a previous study [18]. They also have found that a significant number of variants have changed their classification after the revaluation, so it is important to perform that kind of reassessments in possible or definitive ACM patients. Moreover, they also have focused on the impact that genetic testing has in the ACM diagnosis taking into account the 2010 TFC. However, there are some important differences between the two studies. Their initial classification was not performed systematically following the 2015 ACMG criteria in all patients, only some of them: 53.2% were performed before the 2015 ACMG criteria, whereas 43.8% were performed thereafter. In their study, criteria were significantly less likely to be reclassified in the latter group with 35.1% of variant reclassification. Here, we classified all variants taking into account the 2015 ACMG criteria at the beginning and after 5 years in the same way, with very similar results (30.77 % of change). We propose a limit of time of 5 years to perform the reclassification due to the results that we obtained; after 5 years, a significant number of variants have changed its classification.

VUS variants represented 82.05% of variants in ACM carriers in the original classification in 2016 but only 64.1% of variants in the present study. Moreover, other studies in different cardiomyopathies have similarly concluded that uncertainty is reduced after variant reclassification [23,24]. In the present study, 83.33% of reclassified variants gained certainty, only two of them (DSP c.1696G>A; PKP2 c.2633C>T) have lost certainty changing from LP to VUS. It is important to say that in those two cases, the new data released did not bring uncertainty. A better-performed variant classification may have a major impact in clarifying the origin of ACM in patients as well as in relatives who have borderline phenotypic manifestations. Our genetic results did not impact in clinical diagnosis due to no borderline cases existing in our cohort, either in 2016 or today. It is important to remark that VUS results have been associated with increased patient anxiety, incorrect recall of results, reduced uptake of family screening, and lower rates of sharing information with family [8]. VUS are a remarkable topic also from a forensic point of view, since when they represent the only finding in a negative autopsy case, it is impossible to determine the cause of the death [25]. The fact that VUS represent the majority of the variants found at the genetic testing in cases of sudden cardiac death, is one of the reasons why some authors do not support the mandatory use of post-mortem genetic testing in SCD cases with ambiguous or no macroscopic/microscopic anomalies. However, as shown by this paper, when more information useful to define the significance of the variants is obtained, a noteworthy share of VUS is reinterpreted as P, proving the importance of collecting and sharing data both in clinical and in forensic contexts [26,27,28]. In detail, in our study, four VUS variants changed to LP or P; three of these variants were *PKP2* premature termination codon (PTC), with new global genetic studies identifying no alleles or alleles with an extremely low frequency. Haploinsufficiency in *PKP2* is well established to be disease-causing in ACM [29], so novel rare variants, especially PTC, should be considered as having a potential deleterious role in ACM. Moreover, the other VUS variant that changed into LP (c.1034+2dupT) is an intronic duplication also in *PKP2*.

On the other hand, three other VUS variants in 2016 changed to LB in our reanalysis. In all these cases, massive genotyping data released in the past five years played a key role in reclassification. Therefore, the absolute number of alleles is currently higher than expected for a rare genetic disease such as ACM. Importantly, updated global frequencies allow us to identify data in 53.85% of ACM carriers compared to only 20% of ACM variants identified five years ago. In a recent study, a common reason for genetic reclassification of variants in ACM was a high minor allele frequency in the general population [18].

In addition to updated global frequencies, it is also important to take into account specific scientific findings regarding rare variants to improve knowledge and clarify their role in ACM pathophysiology. In this sense, all rare variants with novel available in vitro and/or family co-segregation data have been reclassified into a higher degree of certainty. In our view, complete family segregation is the most important data to clarify the role of a rare variant, especially if a recessive pattern of inheritance is suspected, as observed in *DSG2*_p.(Thr335Ala) [30]. High variant frequencies in ACM patients (classified as LB in heterozygosis) cannot exclude a pathogenic role if a recessive pattern is suspected, reinforcing the need for cautious personalized interpretation before clinical translation. Several studies demonstrate pathogenesis of recessive mutations in *DSC2* that cause ACM [31,32,33].

Moreover, we should be especially careful in interpreting variants, taking into account suspected disease, affected genes, and previous classification. The *PKP2*_ p.(Met365Val) variant was previously described as potentially disease-causing in Brugada syndrome (BrS) [34]. Although in vitro studies show its effect on the nav1.5 current in a cellular model, its role in ACM is still unclear with no clinical data. Moreover, ECG abnormalities observed in the early stages of ACM may be similar to those in BrS [35,36]. However, the current global frequency of the *PKP2*_ p.(Met365Val) variant has increased during these five years, suggesting a non-deleterious role. Thus, new in vitro studies are also important for reclassifying rare variants into a group with higher certainty. In our study, *DSG2*_ p.(Arg49His) has been reclassified as definitively P based on co-segregation [37] and an in vitro study [38] published in the last 5 years.

Finally, a report has been published recently concerning the role of VUS in inherited arrhythmias and cardiomyopathies [8]. The VUS classification does not provide enough information to make an accurate interpretation, so it is acceptable to consider the variant as “non-actionable” [16,39]. All data conclude that interpretation of a variant classified as a VUS is complex and, sometimes, this result causes a mistaken reduction in familial screenings [40]. It is important to avoid that possibility, so the final objective will be to reduce VUS results as much as possible, although performing segregation studies, when possible, is highly recommended even in VUS variants when there are no more plausible genetic variants to be causal. For this reason, it is appropriate to revaluate variant significance from time to time, at least once every 5 years for ACM-related genes.

## 5. Conclusions

Identifying a definite pathogenic variant is a key point in the diagnosis of ACM. Therefore, accurate interpretation of variants identified in patients should be mandatory. An exhaustive and updated genetic clarification of rare variants associated with ACM is warranted because it has practical consequences for patients and their relatives. Most rare variants remain of ambiguous significance due to lack of functional data, conclusive family segregation, and updated global frequency. It is important to highlight this data because VUS is an uninformative genetic result in translation into clinical practice, and genetic and clinical professionals cannot make informed clinical decisions based on such a classification. We recommend reanalysing rare variants associated with ACM at least once every five years. When significant changes in classification occur, cardiologists should promptly inform interested patients and, if necessary, modify their therapeutic approach.

## 6. Limitations

A different interpretation of genetic data for some rare variants included in our study may induce controversy regarding their role, especially for variants classified as VUS. To minimize controversy, all authors came to a consensus regarding the final classification decision. Importantly, reclassifications in our study should be corroborated in additional larger cohorts of ACM families. Finally, we have not assessed the time or economic cost of reinterpretation.

## Figures and Tables

**Table 1:** **Rare variants found in ACM patients** (grey indicates variants that have been reclassified).

Proband	Diagnosis 2016	Gene	Nucleotide	Protein	dbSNP	ExAC (2016)	GnomAD (2021)	2016 Classification	2021 Classification
1	Possible	*DES*	c.158T>C	p.(Val53Ala)	NI	NI	NI	VUS	VUS
2	Possible	*DSC2*	c.430A>G	p.(Met144Val)	NI	NI	NI	VUS	VUS
3	Definite	*DSC2*	c.2587G>A	p.(Gly863Arg)	rs147109895	6/1,3001 (0.04%)	69/25,1128 (0.02%)	VUS	VUS
4	Definite	*DSG2*	c.146G>A	p.(Arg49His)	rs121913006	NI	1/24,9482 (0.0004%)	LP	P
5	Possible	*DSG2*	c.484delG	p.(Asp162Metfs*10)	rs1158782181	NI	NI	LP	LP
6	Definite	*DSG2*	c.1003A>G	p.(Thr335Ala)	rs191564916	5/1,1919 (0.04%)	130/24,9422 (0.05%)	VUS	LB
7	Possible	*DSG2*	c.1885C>T	p.(Pro629S)	rs200804638	NI	6/24,9336 (0.002%)	VUS	VUS
8	Possible	*DSG2*	c.2825C>T	p.(Thr942Ile)	rs771429752	NI	1/24,8492 (0.0004%)	VUS	VUS
9	Possible	*DSG2*	c.3266G>A	p.(Gly1089Asp)	rs200264407	9/1,2173 (0.07%)	28/24,9268 (0.01%)	VUS	LB
10	Possible	*DSP*	c.130C>T	p.(Arg44Trp)	rs1255744065	NI	2/17,7516 (0.001%)	VUS	VUS
11	Possible	*DSP*	c.559G>T	p.(Val187Phe)	NI	NI	NI	VUS	VUS
12	Possible	*DSP*	c.1063C>T	p.(Gln355*)	rs1561686893	NI	NI	LP	LP
13	Definite	*DSP*	c.1267-2A>G	NI	rs1554106830	NI	NI	LP	LP
14	Possible	*DSP*	c.1297C>T	p.(Arg433Cys)	rs767032884	NI	2/25,1302 (0.0007%)	VUS	VUS
15	Possible	*DSP*	c.1639delC	p.(Leu547Trpfs*8)	NI	NI	NI	LP	LP
16	Possible	*DSP*	c.1696G>A	p.(Ala566Thr)	rs148147581	5/1,3001 (0.03%)	50/25,1036 (0.01%)	LP	VUS
17	Possible	*DSP*	c.2515C>T	p.(His839Tyr)	rs1561693806	NI	1/25,1454 (0.0003%)	VUS	VUS
18	Possible	*DSP*	c.2723G>A	p.(Arg908His)	rs142494121	14/1,2992 (0.1%)	289/25,1322 (0.1%)	VUS	B
19	Possible	*DSP*	c.2723G>T	p.(Arg908Leu)	rs142494121	NI	4/25,1322 (0.001%)	VUS	VUS
20	Possible	*DSP*	c.2867A>G	p.(Asn956Ser)	rs1373071129	NI	1/24,8880 (0.0004%)	VUS	VUS
21	Possible	*DSP*	c.3398A>G	p.(Asp1133Gly)	NI	NI	NI	VUS	VUS
22	Possible	*DSP*	c.3550_3551delCGinsAC	p.(Arg1184Thr)	NI	NI	NI	VUS	VUS
23	Possible	*DSP*	c.3643_3644delAAinsTG	p.(Asn1215Cys)	NI	NI	NI	VUS	VUS
24	Possible	*DSP*	c.8498C>G	p.(Ser2833Cys)	rs767961179	NI	3/24,6912 (0.001%)	VUS	VUS
25	Definite	*JUP*	c.1235C>T	p.(Thr412Met)	rs782551865	NI	2/25,1412 (0.0007%)	VUS	VUS
26	Possible	*PKP2*	c.122C>G	p.(Ala41Gly)	rs1220759009	NI	1/3,1346 (0.003%)	VUS	VUS
27	Definite	*PKP2*	c.259G>C	p.(Val87Leu)	rs750028032	NI	3/25,1410 (0.001%)	VUS	VUS
28	Possible	*PKP2*	c.505A>G	p.(Ser169Gly)	rs139139859	21/1,2979 (0.1%)	294/25,1282 (0.1%)	VUS	B
29	Possible	*PKP2*	c.635G>T	p.(Arg212Leu)	NI	NI	NI	VUS	VUS
30	Possible	*PKP2*	c.1034+2dupT	NI	NI	NI	NI	VUS	LP
31	Possible	*PKP2*	c.1093A>G	p.(Met365Val)	rs143900944	2/1,3004 (0.01%)	67/25,1320 (0.02%)	VUS	LB
32	Definite	*PKP2*	c.1489C>T	p.(Arg497*)	rs151212477	NI	2/21,7850 (0.0009%)	VUS	LP
33	Definite	*PKP2*	c.1643delG	p.(Gly548Valfs*15)	rs794729137	NI	NI	VUS	LP
34	Definite	*PKP2*	c.2104_2111dupTCCTTAGG	p.(Ala705Profs*2)	NI	NI	NI	VUS	LP
35	Possible	*PKP2*	c.2245_2246delGCinsAT	p.(Ala749Ile)	rs1565574704	NI	NI	VUS	VUS
36	Possible	*PKP2*	c.2633C>T	p.(Ser878Phe)	rs1216433436	NI	1/25,1470 (0.0003%)	LP	VUS
37	Possible	*TMEM43*	c.780+3A>G	NI	NI	NI	NI	VUS	VUS
38	Possible	*TMEM43*	c.1026C>G	p.(Asp342Glu)	NI	1/1,3005 (0.007%)	NI	VUS	VUS
39	Possible	*TMEM43*	c.1145T>C	p.(Leu382Pro)	NI	NI	NI	VUS	VUS

NI: allele not identified; VUS, variant of unknown significance; P, pathogenic; LP, likely pathogenic; LB, likely benign; B, benign.

**Table 2:** Most relevant indicators in the reanalyses.

					Indicators for Gene			Indicators for Variant				
		*Nucleotide*	*Protein*	*2021* *Class*	P Gene ^1^ (%) ^1^	P Variant Rate ^2^	Loss of Function ^3^	Population Data (MAF)	Allelic Data ^4^	Hot Spot ^5^	Computational and Predictive Data ^6^	Clinically Reported P before ^7^
**1**	Des	c.158T>C	p.(Val53Ala)	VUS	N/A	N/A	-	NI	NI	Yes	6/13	No
**2**	DSC2	c.430A>G	p.(Met144Val)	VUS	B.T	B.T	-	NI	NI	Yes	1/13	No
**3**	DSG2	c.2587G>A	p.(Gly863Arg)	VUS	B.T	B.T	-	0.02%	69/25,1128	No	10/13	No
**4**	DSG2	c.146G>A	p.(Arg49His)	P	B.T	B.T	-	0.0004%	1/24,9482	Yes	13/13	>10 and no conflict
**5**	DSG2	c.484delG	p.(Asp162Metfs*10)	LP	N/A	N/A	Yes	NI	NI	N/A	N/A	No
**6**	DSG2	c.1003A>G	p.(Thr335Ala)	LB	B.T	B.T	-	0.05%	130/2,4942	Yes	4/13	Unclear
**7**	DSG2	c.1885C>T	p.(Pro629S)	VUS	B.T	B.T	-	0.002%	6/24,9336	No	8/13	No
**8**	DSG2	c.2825C>T	p.(Thr942Ile)	VUS	B.T	B.T	-	0.0004%	1/24,8492	N/A	5/13	No
**9**	DSG2	c.3266G>A	p.(Gly1089Asp)	LB	B.T	B.T	-	0.01%	28/24,9268	No	0/13	No
**10**	DSP	c.130C>T	p.(Arg44Trp)	VUS	B.T	B.T	-	0.001%	2/17,7516	Yes	6/13	No
**11**	DSP	c.559G>T	p.(Val187Phe)	VUS	B.T	B.T	-	NI	NI	Yes	4/13	No
**12**	DSP	c.1063C>T	p.(Gln355*)	LP	N/A	N/A	Yes	NI	NI	N/A	N/A	No
**13**	DSP	c.1267-2A>G	NI	LP	N/A	N/A	Yes	NI	NI	N/A	Moderate	No
**14**	DSP	c.1297C>T	p.(Arg433Cys)	VUS	B.T	B.T	-	0.0007%	2/25,1302	Yes	10/13	No
**15**	DSP	c.1639delC	p.(Leu547Trpfs*8)	LP	N/A	N/A	Yes	NI	NI	N/A	N/A	No
**16**	DSP	c.1696G>A	p.(Ala566Thr)	VUS	B.T	B.T	-	0.01%	50/25,1036	Yes	0/13	No
**17**	DSP	c.2515C>T	p.(His839Tyr)	VUS	B.T	B.T	-	0.0003%	1/25,1454	Yes	4/13	No
**18**	DSP	c.2723G>A	p.(Arg908His)	B	B.T	B.T	-	0.10%	289/25,1322	Yes	8/13	No
**19**	DSP	c.2723G>T	p.(Arg908Leu)	VUS	B.T	B.T	-	0.001%	4/25,1322	Yes	7/13	No
**20**	DSP	c.2867A>G	p.(Asn956Ser)	VUS	B.T	B.T	-	0.0004%	1/24,8880	Yes	3/13	No
**21**	DSP	c.3398A>G	p.(Asp1133Gly)	VUS	B.T	B.T	-	NI	NI	No	6/13	No
**22**	DSP	c.3550_3551delCGinsAC	p.(Arg1184Thr)	VUS	B.T	B.T	-	NI	NI	No	N/A	No
**23**	DSP	c.3643_3644delAAinsTG	p.(Asn1215Cys)	VUS	B.T	B.T	-	NI	NI	No	N/A	No
**24**	DSP	c.8498C>G	p.(Ser2833Cys)	VUS	B.T	B.T	-	0.001%	3/24,6912	Yes	8/13	No
**25**	JUP	c.1235C>T	p.(Thr412Met)	VUS	B.T	B.T	-	0.0007%	2/25,1412	No	8/13	No
**26**	PKP2	c.122C>G	p.(Ala41Gly)	VUS	A.T	A.T	-	0.003%	1/3,1346	No	5/13	No
**27**	PKP2	c.259G>C	p.(Val87Leu)	VUS	A.T	A.T	-	0.001%	3/25,1410	No	9/13	No
**28**	PKP2	c.505A>G	p.(Ser169Gly)	B	A.T	A.T	-	0.10%	294/25,1282	No	12/13	No
**29**	PKP2	c.635G>T	p.(Arg212Leu)	VUS	A.T	A.T	-	NI	NI	No	5/13	No
**30**	PKP2	c.1034+2dupT	NI	LP	N/A	N/A	Yes	NI	NI	N/A	N/A	No
**31**	PKP2	c.1093A>G	p.(Met365Val)	LB	A.T	A.T	-	0.02%	67/25,1320	No	2/13	No
**32**	PKP2	c.1489C>T	p.(Arg497*)	LP	A.T	A.T	Yes	0.0009%	2/21,7850	N/A	N/A	1
**33**	PKP2	c.1643delG	p.(Gly548Valfs*15)	LP	N/A	N/A	Yes	NI	NI	N/A	Very Strong	>17 and no conflict
**34**	PKP2	c.2104_2111dupTCCTTAGG	p.(Ala705Profs*2)	LP	N/A	N/A	Yes	NI	NI	N/A	N/A	No
**35**	PKP2	c.2245_2246delGCinsAT	p.(Ala749Ile)	VUS	A.T	A.T	-	NI	NI	No	N/A	No
**36**	PKP2	c.2633C>T	p.(Ser878Phe)	VUS	A.T	A.T	-	0.00030%	1/25,1470	No	11/13	No
**37**	TMEM43	c.780+3A>G	NI	VUS	N/A	N/A	No	NI	NI	No	N/A	No
**38**	TMEM43	c.1026C>G	p.(Asp342Glu)	VUS	B.T	B.T	-	NI	NI	No	2/13	No
**39**	TMEM43	c.1145T>C	p.(Leu382Pro)	VUS	B.T	B.T	-	NI	NI	No	4/13	No

Criteria and threshold based on Varsome. In grey meets criteria for pathogenicity. ^1^ Pathogenic non-VUS missense variants in gene (%). ^2^ Missense variant in a gene with a low rate of benign missense variation and in which missense variants are a common mechanism of disease.^3^ Loss of function is a well-established disease mechanism (premature termination codon (PTC) only). ^4^ Allele count for dominant gene threshold. ^5^ Located in a mutational hot spot and/or critical and well-established functional domain. ^6^ Computational evidence (conservation, evolutionary, splicing impact, etc.) using 13 in silico predictors for missense variants. AutoPVS1 used for intronic variants. ^7^ Reputable source recently reported variant as pathogenic in probands with consistent phenotypes (P). B.T, below threshold; A.T, above threshold; NI, allele not identified; N/A, data not available or variant does not meet criteria to apply indicator; MAF, minor allele frequency; VUS, variant of unknown significance; P, pathogenic; LP, likely pathogenic; LB, likely benign.
